# Comment on: “Melanisation of *Aspergillus terreus*—Is Butyrolactone I Involved in the Regulation of Both DOPA and DHN Types of Pigments in Submerged Culture? Microorganisms 2017, 5, 22”

**DOI:** 10.3390/microorganisms5020034

**Published:** 2017-06-21

**Authors:** Elena Geib, Matthias Brock

**Affiliations:** Fungal Genetics and Biology, School of Life Sciences, University of Nottingham, University Park, NG72RD Nottingham, UK

## Abstract

A recent article by Palonen et al. describes the effect of butyrolactone I on the expression of a secondary metabolite biosynthesis gene cluster from *Aspergillus terreus* that shows similarities to fusarubin biosynthesis gene clusters from *Fusarium* species. The authors claim that two different types of pigments are formed in *Aspergillus terreus* conidia, whereby one pigment is termed a DOPA-type melanin and the second a DHN-type melanin. Unfortunately, the terminology of the classification of melanin-types requires revision as Asp-melanin present in *A. terreus* conidia is clearly distinct from DOPA-melanins. In addition, some hypotheses in this manuscript are based on questionable data published previously, resulting in incorrect conclusions. Finally, as biochemical data are lacking and metabolite production is only deduced from bioinformatics and transcriptomic data, the production of a second pigment type in *A. terreus* conidia appears highly speculative.

Palonen et al. studied the expression of a newly identified *pgm* gene cluster from *Aspergillus terreus* during growth in submerged culture conditions and in the presence or absence of the quorum sensing metabolite butyrolactone I [1]. Transcriptional analyses revealed that in the presence of butyrolactone I the expression of *pgm* cluster genes increase with cultivation time. Simultaneously, expression of the two genes, *melA* and *tyrP*, responsible for Asp-melanin biosynthesis in *A. terreus* conidia [2] decrease in the presence of butyrolactone I. Since butyrolactone I increases sporulation of *A. terreus* in submerged cultures [3] and the *pgm* gene cluster shows similarity to perithecium pigment biosynthesis gene clusters from *Fusarium* species [1], the authors conclude that besides the well-characterised Asp-melanin, a dihydroxynaphtalene-type melanin (DHN-melanin) might be produced in *A. terreus* conidia. This speculation additionally is based on a manuscript by Pal et al. [4], in which inhibitor studies indicated that *Aspergillus* species simultaneously produce a DHN-melanin and an l-DOPA-melanin as conidia pigments. Unfortunately, the study by Pal et al. [4] lacked an investigation of solvent controls on conidia pigment formation, which would have revealed that inhibitor studies need to be interpreted with care. Kojic acid, an inhibitor of the l-DOPA-melanin pathway, was solved in 70% DMSO and applied to growth media. Analysis of conidia melanisation revealed that most *Aspergillus* species lost colouration in the presence of kojic acid, which led to the conclusion that an l-DOPA-melanin pathway may be involved in pigmentation of conidia [4]. As can be seen in Figure 1 of this letter, DMSO rather than kojic acid is responsible for this loss in pigmentation with the highest effect on *Aspergillus fumigatus*, followed by *Aspergillus nidulans* and *Aspergillus niger*. No effect was observed on *A. terreus*. Similarly, the laccase inhibitor pyrolidone, which inhibits polymerisation of DHN-melanin precursors, showed a strong effect on *A. fumigatus*, followed by *A. nidulans* and *A. niger*. Again, no effect is observed on *A. terreus*. This indicates that the inhibitory effect caused by kojic acid may eventually be caused by the solvent and does not confirm an l-DOPA-melanin pathway in *Aspergillus* species. Furthermore, these experiments confirm that *A. terreus* shows no sensitivity against either type of melanin biosynthesis inhibitors, which is in agreement with the novel type of Asp-melanin produced by this fungus.

However, Palonen et al. [1] denote the formation of Asp-melanin in *A. terreus* as a type of DOPA-melanin. The authors base this on the fact that a tyrosinase is involved in its biosynthesis. Unfortunately, we cannot agree with this definition. l-DOPA (l-dihydroxyphenylalanine) is not a precursor of Asp-melanin. 4-Hydroxyphenylpyruvate is the substrate for the NRPS-like enzyme MelA that forms the precursor aspulvinone E [2,5]. The subsequent hydroxylation of aspulvinone E by the tyrosinase TyrP leads to auto-polymerisation of aspulvinone E units [2]. Reaction intermediates have been analysed and no l-DOPA has been detected at any step as precursor molecule [2]. Furthermore, the tyrosinase TyrP does not use l-DOPA as substrate. Therefore, defining Asp-melanin as an l-DOPA-type melanin is not correct, as Asp-melanin is a novel type of melanin pigment not related to either l-DOPA- or DHN-melanin.

As deletion of *melA* results in white conidia [2,5] and in vitro studies with purified MelA and TyrP proteins have reconstituted the Asp-melanin biosynthesis pathway, it can be excluded that an additional DHN-melanin is produced in *A. terreus* conidia, at least, when grown on solid media or as biofilm surface cultures in liquid media. Palonen et al. [1] used a submerged culture condition throughout their studies in which they supplemented the medium artificially with butyrolactone I. Under these conditions, an increased expression of the *pgm* gene cluster accompanied by a decreasing expression of the Asp-melanin gene cluster was observed. As *pgm* cluster expression further increased at the late stage of fermentation and, furthermore, the culture turned into an increasingly brown colour after nine days of incubation, the authors speculated that a DHN-melanin type pigment deriving from the *pgm* gene cluster might be formed under these cultivation conditions.

However, this speculation has several problems: (i) No analysis of the type or number of spores has been presented. *A. terreus* not only produces conidia from conidiophores, but also accessory conidia, which are generally hyaline. Which types of spores and in which numbers have been produced under these conditions? What colour do they show? By which experiments did the authors exclude that the brownish pigment does not derive from Asp-melanin? Is the brownish colour also observed in a *melA*^-^ mutant? (ii) No data on secondary metabolite profiles have been presented. On which basis other than bioinformatics do the authors base their assumption of a DHN-melanin formed by *A. terreus* under these culture conditions? The authors may be aware that most fungal polyketide synthases (PKS) are iterative and, currently, it appears impossible to predict the number of malonyl-CoA extender units used by such an enzyme. The similarity of a fungal PKS to other PKSs may provide a hint towards its product, but without experimental data it remains purely speculative as to which kind of product is formed.

Interestingly, the authors do not seem to consider that the gene cluster identified in this study may be involved in production of an ascospore pigment. Although butyrolactones are naturally produced by *A. terreus* [3], the conditions with external supplementation of media with butyrolactone I appear rather artificial and may not resemble the natural induction conditions of the *pgm* gene cluster. However, as a quorum sensing molecule butyrolactones cannot be excluded to play a role in sexual reproduction of *A. terreus*. As the *pgm* gene cluster identified here shows highest similarity to gene clusters producing pigments in fungal perithecia—which are sexual reproduction structures of ascomycetes—it is difficult to understand why the authors did not follow this hypothesis further.

In conclusion, while the manuscript by Palonen et al. [1] shows solid transcriptional analyses on the *pgm* gene cluster from *A. terreus*, all hypotheses on the nature of the resulting metabolite are purely speculative and should be taken as such. Furthermore, the definition of Asp-melanin as an l-DOPA type melanin needs revision.

## Figures and Tables

**Figure 1:**
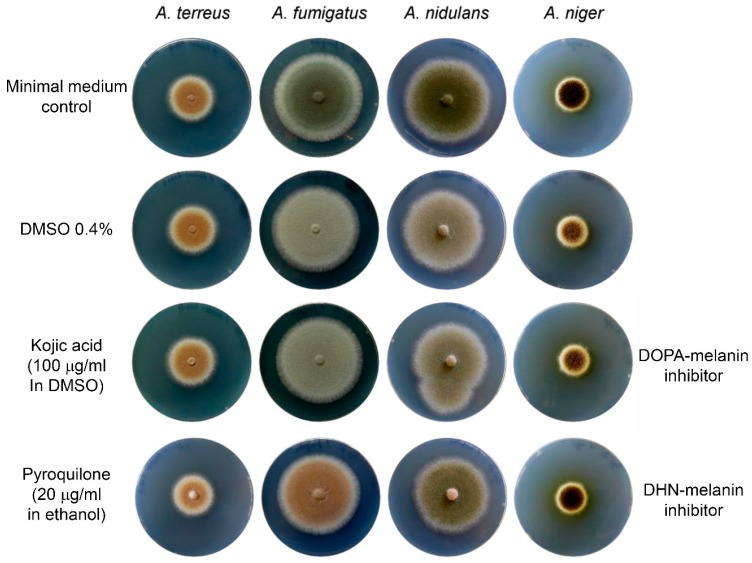
Effect of DMSO, kojic acid and pyroquilone on conidia pigmentation of different *Aspergillus* species. The following strains were used: *A. terreus* SBUG844 (Jena Microbial Resource Collection, HKI Jena, Germany), *A. fumigatus* CBS144.89 (CBS-KNAW Collection, Utrecht, Netherlands), *A. nidulans* FGSC A4 and *A. niger* FGSC A1144 (FGSC = Fungal Genetics Stock Center, Kansas, USA). All strains were grown in glucose containing minimal media. DMSO strongly affects conidia pigmentation of all *Aspergillus* species except *A. terreus*, which makes the assessment of the effect of kojic acid on conidia pigmentation (solved in DMSO) difficult. The DHN-melanin inhibitor pyroquilone (solved in ethanol) inhibits pigment polymerisation of *A. fumigatus*, and, at the applied concentration, shows weak effects on *A. nidulans* and *A. niger*, but not *A. terreus*. Growth in the presence of 0.5% ethanol does not affect conidia pigmentation of any strain (not shown).
